# Editorial: miRNAs and their role in endocrine cancer progression: from prognosis to treatment

**DOI:** 10.3389/fendo.2023.1257692

**Published:** 2023-08-18

**Authors:** Prem Prakash Kushwaha, Francesco Doglietto, Gianluca Baldanzi

**Affiliations:** ^1^ School of Medicine, Case Western Reserve University, Cleveland, OH, United States; ^2^ Neurosurgery, Università Cattolica del Sacro Cuore, Rome, Italy; ^3^ Neurosurgery, Fondazione Policlinico Universitario A. Gemelli Istituto di Ricovero e Cura a Carattere Scientifico (IRCCS), Rome, Italy; ^4^ Department of Translational Medicine, University of Piemonte Orientale, Novara, Italy; ^5^ Center for Translational Research on Allergic and Autoimmune Diseases (CAAD), University of Piemonte Orientale, Novara, Italy

**Keywords:** oncology, gene expression, post-transcriptional regulation, gene silencing, biomarker, interfering RNA

MicroRNAs (miRNAs) constitute a key regulatory component of heterogeneous RNA world that mediates the post-transcriptional regulation of gene expression. By binding to messenger and non-coding RNAs, miRNAs control gene expression and create a regulatory layer of interactions between different transcripts. Recently, two main driving forces propelled research in this field: i) due to their central role in cell homeostasis, microRNAs are obviously perturbed in pathologies such as tumors; ii) RNA-based therapeutics are entering the drug market, making this regulatory layer amenable to experimental and potentially therapeutic targeting.

This Frontiers in Endocrinology Research Topic focuses on the role of miRNAs in endocrine cancer, looking at them as biomarkers for diagnosis and prognosis and as protagonists of mechanisms driving cell transformation. This is an active field of research, as evidenced by the Thakur and Thakur systematic review “*The Interplay of Sex Steroid Hormones and MicroRNAs in Endometrial Cancer: Current Understanding and Future Directions*”, which investigates the dysregulation of miRNA in endometrial cancer, a hormone-dependent malignancy. They specifically focused on miRNA-mediated regulation of sex steroid hormone signaling, which is crucial for the pathogenesis of this cancer type. The review explains how miRNA works in endometrial cancer and identifies possible new targets for treatment. It also notes that the miRNA expression profile can predict how a patient will respond to hormone therapy (Thakur and Thakur).

The role of miRNAs as biomarkers in endocrine cancers is a mainstream argument in research, as evidenced by three Research Topic articles.

Niedra et al. in “*Case Report: Micro-RNAs in Plasma From Bilateral Inferior Petrosal Sinus Sampling and Peripheral Blood From Corticotroph Pituitary Neuroendocrine Tumors*” explore the pattern of circulating miRNAs in plasma close to pituitary neuroendocrine tumors after the corticotropin-releasing hormone administration. The researchers discovered 49 distinct miRNAs that were differentially expressed, five of which were differentially expressed at all three-time intervals. To determine the correlation between modulated miRNA and disease, they analyzed the levels of two highly modulated miRNAs (miRNA-7-5p and miRNA-375-3p) in pre- and post-operative samples, in addition to tumor tissue. According to their research, a miRNA signature could serve as a diagnostic marker for this type of tumor (Niedra et al.).

In their research article titled “*Construction of a serum diagnostic signature based on m5C-related miRNAs for cancer detection*”, Tang et al. go further, as they created a serum diagnostic signature for detecting multiple types of cancer based on miRNAs related to 5-methylcytosine. Such a signature displayed excellent accuracy, was unaffected by patient age, sex, or noncancerous disease, and displayed satisfactory performance for distinguishing tumor types. The diagnostic performance of the miRNA signature was superior to that of conventional tumor biomarkers in early-stage cancers, suggesting a putative use for large-scale cancer screening programs (Tang et al.).

Xie et al., in “*The Potential Role of CDH1 as an Oncogene Combined with Related miRNAs and Their Diagnostic Value in Breast Cancer*”, explore cadherin 1 expression in breast cancer and the miRNA network regulating its expression. Their data support a function of miR-20a in regulating tumor cell stemness through the modulation of cadherin 1, a key molecule in breast cancer. Moreover, soluble E-cadherin and miR-20a in serum are found to be noninvasive markers for breast cancer diagnosis (Xie et al.).

Finally, the involvement of miRNAs in the mechanisms of cell transformation is the focus of Al-Sisan et al. in “*Differential miRNA Expression of Hypoxic MCF7 and PANC-1 Cells*”, where two popular cell models of breast and pancreatic cancer are maintained in hypoxic conditions before miRNA quantification to highlight modulated ones, gain insight into pathology and identify novel therapeutic targets. They correlated miR-221, miR-21, miR-155, and miR-34 alterations in PANC-1, and miR-93, miR-20a, miR-15, and miR-17 in MCF7 with the emergence of an epithelial-to-mesenchymal transition-like phenotype, alterations in proliferation rates, and doxorubicin resistance (Al-Sisan et al.).

By joining research articles and revisions of the literature, we aim to sum up current knowledge and propel further advancements, suggesting that non-coding RNA, particularly miRNAs, are major players in endocrine organs and endocrine-related disorders. In the cancer field, a better understanding of pathogenic mechanisms, new therapeutic targets, and improved biomarkers are a stringent necessity, and the works presented in this Frontiers in Endocrinology Research Topic indicate that miRNA may well contribute to this.

## Author contributions

GB: Writing – original draft. PK: Writing – review & editing. FD: Writing – review & editing.

